# Review of Batteryless Wireless Sensors Using Additively Manufactured Microwave Resonators

**DOI:** 10.3390/s17092068

**Published:** 2017-09-09

**Authors:** Muhammad Usman Memon, Sungjoon Lim

**Affiliations:** School of Electrical and Electronics Engineering, College of Engineering, Chung-Ang University, 221 Heukseok-dong, Dongjak-gu, Seoul 156-756, Korea; musmanm@outlook.com

**Keywords:** inkjet printing, 3D printing, screen printing, RF sensors, wireless, RF resonators

## Abstract

The significant improvements observed in the field of bulk-production of printed microchip technologies in the past decade have allowed the fabrication of microchip printing on numerous materials including organic and flexible substrates. Printed sensors and electronics are of significant interest owing to the fast and low-cost fabrication techniques used in their fabrication. The increasing amount of research and deployment of specially printed electronic sensors in a number of applications demonstrates the immense attention paid by researchers to this topic in the pursuit of achieving wider-scale electronics on different dielectric materials. Although there are many traditional methods for fabricating radio frequency (RF) components, they are time-consuming, expensive, complicated, and require more power for operation than additive fabrication methods. This paper serves as a summary/review of improvements made to the additive printing technologies. The article focuses on three recently developed printing methods for the fabrication of wireless sensors operating at microwave frequencies. The fabrication methods discussed include inkjet printing, three-dimensional (3D) printing, and screen printing.

## 1. Introduction

Progress in the area of wireless communication is directed toward a nonstop improvement of device operations, ubiquity, and commercial viability of devices. Evolving research into millimeter wave (mm-wave) radio frequency (RF) communication operations at frequencies ranging 30–300 GHz is important for the advancement of such automation as gigabit wireless local area networks, automobile contact prevention, self-operational steering radar systems, and fine-quality beam-tuning image-processing equipment. The classic production methods for mm-wave structures comprise patterning, lithographic masking, and reproduction of materials that require the use of severe liquid materials, and are expensive. In order to enable the maintained assimilation and spread of rising mm-wave methodologies, attempts should be made to boost their adaptability and minimize the price of component manufacture.

An additive electronic fabrication technique known as “inkjet printing” has been gathering immense attention for industrial uses as a greatly scalable, cost-effective, and above all, environment-friendly substitute to classical lamination-based fabrication methods [1]. Using thick/thin polymer-based and conductive nanoparticle-based ink materials, multiple layering structures for RF industry—e.g., fully printed transformers, inductors and capacitors [2,3,4]—have been realized, using inkjet-printing on stretchy materials. Through the refinement and characterization of these ink materials, multilayer RF structures operational in the millimeter-wave frequency range have been accomplished with the help of inkjet-printing manufacture on both solid and stretchy materials [5,6]. Conversely, numerous latest presentations of inkjet-printed millimeter-wave modules suffer from restrictions existing in the distinctive multiple layers of RF structure designs—e.g., material properties and uniform thinness of corporate laminating materials—along with difficulties in multiple layers of laminating material treatment (e.g., material alignment, bonding, and stacking) [7,8,9,10]. The upright assimilated, additive aspect of inkjet-printed structure in electronics allows the integration with structures of significant interest, such as system-on-chip modules, stretchable and wearable electronics, and conformable/reconfigurable/rollable configurations [11,12]. With the help of this integration, the versatility and efficiency of RF millimeter-wave systems has drastically increased by directly post-process depositing antennas into any solid or stretchable active circuit configuration.

There are many additive manufacturing techniques such as gravure/dispenser printing, spray deposition, stereolithographic, polymer-jet fused deposition modeling, laminated object manufacturing, selective laser sintering, electronic beam melting containing liquid, solid and powder based processes [13]. Nevertheless, the most popular additive manufacturing techniques for microwave printed electronics are 3D printing, inkjet printing, and screen printing. Therefore, we review these three additive printing techniques for microwave resonator-based wireless sensor applications. 

In the case of creating the preferred designs for malleable electronic devices, many recent material processing methods have been proposed including lithographic [14,15], stencil printing [16,17,18,19,20,21], microchannel molding and coating, and space-filling techniques [22,23]. However, the aforementioned techniques are expensive, lack fabrication scalability, or employ complex procedures and, consequently, their widespread implementation has been restricted. Furthermore, three-dimensional (3D) printing does not suffer from these restrictions, and has started to become a mainstream additive production technique. This technique uses the mechanism of constructing complex-shaped objects layer-by-layer containing thorough digital outlines [24]. Further, 3D printing technology has many benefits, such as shorter fabrication time and a capability to construct multifarious configurations and shapes with multiple materials at the same time, which certainly is beyond the capabilities of conventional fabrication processes. 

The speedy evolution of 3D and four-dimensional (4D) (time-managing) printing machinery promises exclusive rewards in the formation of optimum non-orthogonal shapes, on-demand deposition of nanostructures and other RF modules, such as antennas, and multiple layers on stretchy hermetic RF suites for flexible electronics. However, there are noteworthy obstacles in the construction of fully 3D multiple layer configurations. One of these obstacles is the bridging or overhanging complications, where the materials are affected by gravity throughout the printing course. Consequently, most schemes attempt to circumvent extremely overhung 3D structures and approve printing electrical components over a surface, such as those described in [18,25]. To be precise, these systems belong to the 2.5D printing classification. The existing marketplace proposes three solutions for printing extreme overhang structures: zero support, solid support structures, and non-solid supporting structures. Zero support printing is in the early stages of development [26]. Consequently, many limitations in the selection of printing materials and geometry design still exist. Solid support structures are the most common solution for extreme overhang designs. However, printing in this manner wastes material. Moreover, this technique is time-consuming, and it is difficult to remove the support. Consequently, small marks, which cause dimensional inaccuracies, remain on the printed object. In the case of non-solid supporting structures, powder-based technologies such as selective laser sintering and direct metal laser sintering are extensively adopted printing methods for commercial use. These techniques can use the surrounding powder to hold the structure in place during printing; e.g., the 3DPandoras printer [27]. However, the powder can be tough to remove, particularly when printing nylon or metal. Other methods in early development, such as embedded 3D printing [28], are still not sufficiently developed to be used in fabrication, since the ink utilized requires a high resistivity (117 Ωcm) and the printing process is complicated.

Screen printing is the most popular and mature technology for printed electronics, since it has been employed in the electronics industry to print metallic interconnects on printed circuit boards for a long period. This technique is faster and more versatile than other printing tools, since the fabrication process is simple, affordable, quick, and adaptable. Screen-printed devices can be reproduced by repeating a few steps, and an optimum operating envelope can be developed quickly [29,30,31,32,33,34]. The feasibility of screen printing for flexible electronics has been demonstrated through the production of many printed sensors, electronic devices, and circuits. For example, all-screen-printed thin-film transistors (TFTs) have been demonstrated in [33,35,36]. Screen printing has been used to develop organic light-emitting diodes, following the investigation of the fabrication process and parameters of the screen printing solution i.e., viscosity of the solution and mesh count of the screen [37]. Multilayer high-density flexible electronic circuits, connected to embedded passive and optical devices through micro via holes, have been realized using advanced screen printing processes [32]. Screen printing is also used for patterning to develop shadow masks for the fabrication of organic TFTs. Screen-printed electrical interconnects for a temperature sensor on a polyethylene terephthalate (PET) substrate have been reported in [38].

However, in this review article, a summary/review of the developments in only printing technologies such as inkjet, 3-D and screen printing for the fabrication of batteryless wireless sensors operating at microwave frequencies is presented. Easier processing stages, minimized material waste, high speed and cost-effective substances, and simple patterning procedures render printing tools very attractive for accurate, multi-layered, and cost-effective development [39]. These features of printed electronics have allowed researchers to explore new avenues for material processing and to develop sensors and systems on non-planar surfaces that are otherwise difficult to realize with the conventional wafer-based fabrication techniques. In this paper, we compare the current developments in the three aforementioned additive printing fabrication techniques, with respect to their fabrication time, power consumption, and complexity.

The design and analysis of inkjet-printed sensors are presented in Section 2. Section 3 focuses on the construction of a 3D RF sensor and its technological advantages. Section 4 comprises a review of the latest screen-printed sensors. Section 5 compares the technologies presented and summarizes this review.

## 2. Inkjet Printed Sensors

This section describes the advanced inkjet-printed batteryless RF sensor devices. Inkjet-printed wireless sensor systems for numerous future applications are introduced in terms of their environmental impact and performance as a sustainable technology.

### 2.1. Inkjet-Printing on Paper Material

Inkjet-printing methods have many benefits for RF sensor fabrication. Inkjet printing technology is cost-effective and environment-friendly because no hazardous chemicals are used to wash away the unwanted metals on the surface of a substrate. In this technique, nanoparticle ink is deposited at the desired position. Consequently, there are no by-products because inkjet printing is an additive fabrication method. The advantages of this technology, such as fast fabrication and ease of mass production, also reduce the cost of inkjet-printed electronics. The electrical properties of inkjet-printed silver nanoparticles were thoroughly studied in [1]. Many microwave applications utilizing silver nanoparticles have been proposed in [40,41,42]. The conductivity of the inkjet printing silver nanoparticle inks is approximately 1.12 × 10^7^ S/m, which is sufficiently high for microwave or millimeter-wave applications.

Inkjet printing technology can be used to fabricate various electronic devices on many kinds of materials; consequently, it is possible to utilize an environment-friendly alternative, such as paper, as a substrate. Paper is a very attractive substrate in inkjet-printed electronics for agricultural applications. Paper is a low-cost, renewable, and inkjet printable material. Apart from being one of the cheapest materials in the world, paper decomposes completely in agricultural environments. Moreover, there are many kinds of paper, such as hydrophobic, porous, and translucent paper. The hydrophilic property of normal paper is useful for implementing humidity or rainfall sensors. These sensors are widely adopted because water monitoring is crucial in agriculture. The properties of paper that are useful for inkjet printing have been reported in many studies based on measurements using different characterization techniques, such as ring resonator or T-resonator methods [1,40]. Paper has a reported dielectric constant ($\varepsilon_{r}$) of approximately 3.0 and a loss tangent (tan δ) of approximately 0.05–0.06. The relatively high loss of paper is not a critical issue for radio frequency identification (RFID) or planar structures, which have a low Q-factor, because paper is very thin. This high loss results in low interaction between the electric field (E-field) and paper substrate.

### 2.2. RFID Enabled Sensor, Retro-Directive Transponders, and an Inkjet-Printed Sensor Platform

#### 2.2.1. RFID-Empowered Sensor

RFID-empowered sensor devices have many benefits over state-of-the-art sensor components in terms of cost-effectiveness and ease of use. Typically, the price of an RFID tag is small and the structure is comparatively simple (reader and sensor tag). Thus, it is possible to realize an RFID-empowered sensor device over a large agricultural field at a low cost. RFID principles are also appropriate for current wireless sensor networks, which are easy to implement [43].

**Figure 1:**
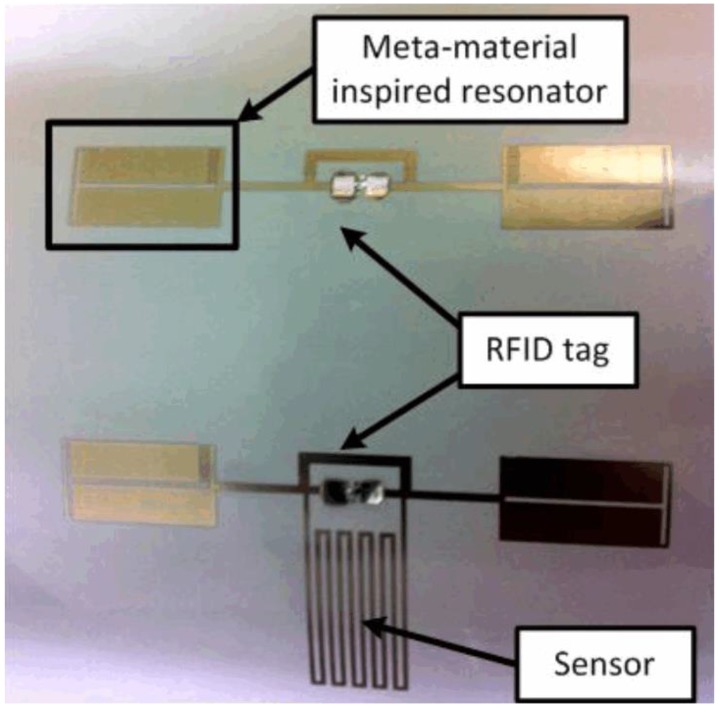
Inkjet-printed capacitive sensor [44].

In this subsection [44], an inkjet-printed RFID-empowered sensor device for haptic and water-level recognition is proposed. The device contains two similar RFID tags for the ultra-high-frequency (UHF) band at approximately 915 MHz. The proposed sensor is combined with one of the RFID tags. The sensor is a meandering line with a self-resonant frequency of approximately 915 MHz. When a material with a dielectric constant and loss tangent different from those of air comes into contact with the meandering stripline, the capacitance of the component varies, which results in shifting of the resonating frequency of an RFID tag. Since the RFID tags have identical resonant frequencies, their unique IDs are returned at a similar frequency values when they are activated by the tag reader. However, the resonant frequency of the antenna connected to the sensor device (Figure 1) is moved to a lower frequency when the detector is in contact with human skin or liquid (water in this case). Utilizing the tag without the sensor for a reference, the existence of an analyst can be easily determined. Similarly, the level of water can be identified because the variation of the capacitance of the sensor device influences the resonant frequency of the RFID-empowered structure. A metamaterial-empowered resonating element is attached to the tags for overturning crosstalk between the two tags.

#### 2.2.2. Retro-Directive Transponder for Sensing

**Figure 2:**
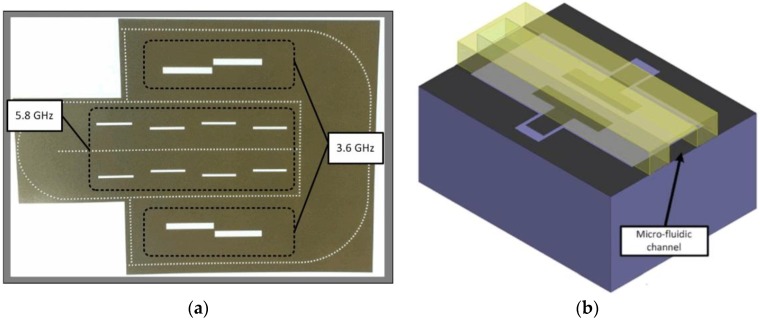
(**a**) Inkjet-printed, dual-band, substrate-integrated waveguide retro-directive array on paper; and (**b**) proposed microfluidic sensor [47].

A retro-directive antenna array can re-transmit an interrogation signal to its source without any complicated computations [45]. The Van Atta topology is a widely used retro-directive antenna array topology owing to its simple structure and passive implementation [46]. The integration of a microfluidic sensor with a retro-directive antenna array was proposed in [47,48]. The resulting device is purely passive and has a self-steering capability. The self-steering capability results in strong system performance owing to the wide readable angle of the passive transponder. This property is particularly critical in radar cross-section (RCS)-based backscattering communication applications, such as passive wireless sensor systems. For example, the back-scattered power of most passive RCS-based wireless sensors depends on the illumination angle. Retro-directive antenna arrays can be used to improve the performance of the sensor because these antenna arrays can reflect near-identical powers to the interrogation direction over a broad angle. The proposed inkjet-printed, dual-band, substrate-integrated waveguide retro-directive array, and microfluidic sensor are shown in Figure 2 [47]. The operation of this device suggests a potential application as a chipless RFID-enabled sensor tag operating at two different frequencies for temperature or water quality sensing. The variation of the RCS of the microfluidic sensor can be measured over a broad range owing to the retro-directive transponder. The dual-band property of the retro-directive transponder results in the ability to sense two targets at two different frequencies.

#### 2.2.3. Inkjet-Printed Sensor Platform

**Figure 3:**
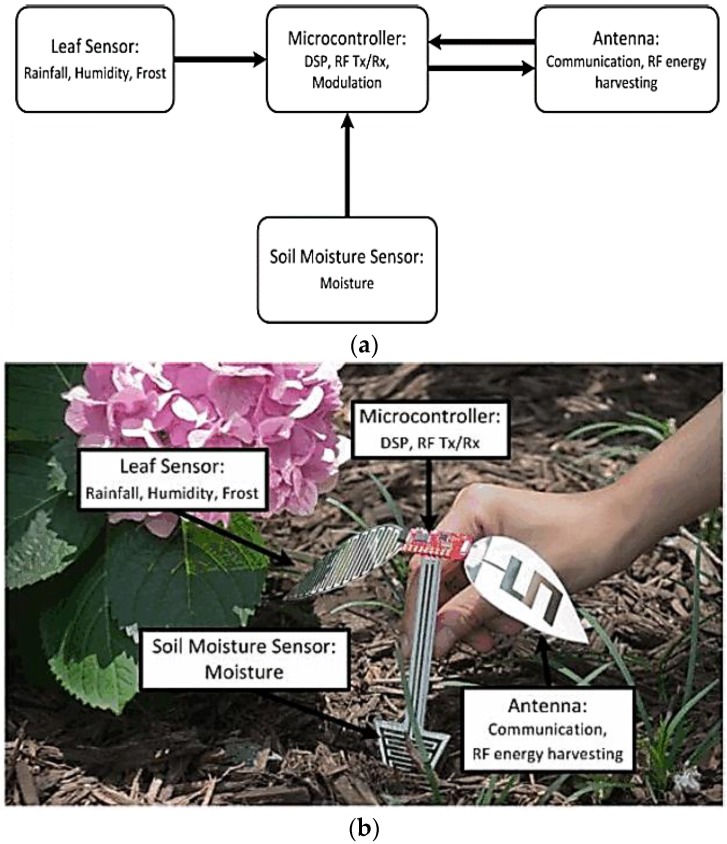
Inkjet-printed sensor platform for agriculture applications: (**a**) block diagram of system; and (**b**) proposed design in operation [49].

A cost-efficient inkjet-printed sensor module for agricultural applications was recently proposed in [49]. The sensor platform has been improved to detect ambient moisture content, water content of the soil, and rainfall because moisture sensing is a crucial aspect of farming. The block diagram of the system, which contains a leaf sensor, soil humidity sensor, microcontroller unit, and antenna, is shown in Figure 3a. The capacitance of the leaf sensor and soil humidity sensor differ based on the water content and humidity of the soil or the environment surrounding the sensor module. The microcontroller detects variations in the capacitance of the leaf sensor and the soil moisture sensor. The microcontroller processes the data collected from the sensor and broadcasts this information through the antenna. The microcontroller and antenna can also be used to gather ambient power information to initialize the microcontroller or reduce battery consumption [50]. In contrast to traditional sensor modules, all passive elements are inkjet-printed on an eco-friendly paper substrate. Finally, dense monitoring of rainfall and soil humidity over large agricultural fields is possible owing to the advantages of inkjet printing technology, such as low fabrication cost and ease of mass production. An implementation of the sensor platform is shown in Figure 3b. The soil moisture sensor is buried in the ground to detect surface soil humidity. The leaf sensor, microcontroller, and antenna are visible. The uncovered constituents can be chemically layered with Parylene or silicone, if required, to extend the lifetime and protect the sensor platform.

### 2.3. A Fully Inkjet-Printed Wireless and Chipless Sensor for Carbon Dioxide (CO*_2_*) and Temperature Detection

**Figure 4:**
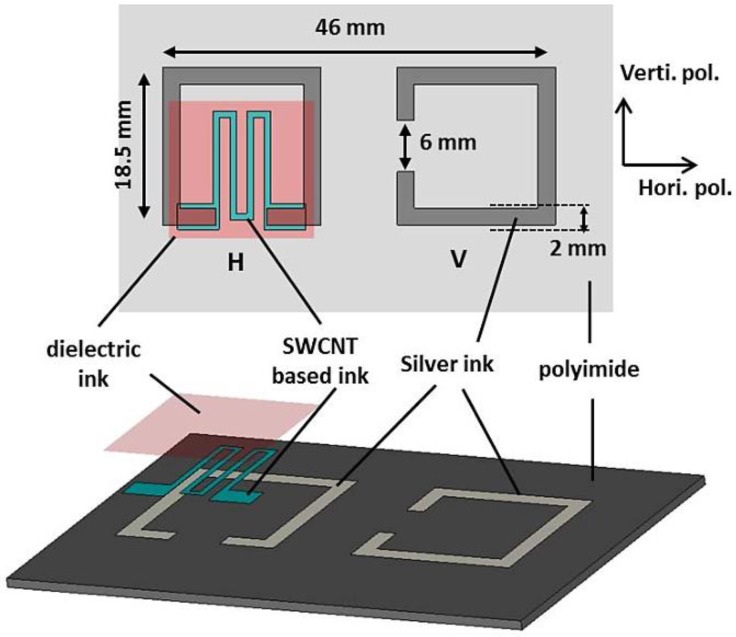
Top view and side view of the fully inkjet-printed dual-polarized sensor device fabricated using three different inks [51].

This subsection describes a printed CO_2_ and temperature sensor, which utilizes different industrial inks. The sensitivity of a batteryless detector or sensor is the result of the removal of complex single-walled/polymer carbon-nanotube (SWCNT) ink material [51]. It was recently demonstrated that graphene sheets and carbon nanotubes (CNT) provide robust sensitivity to several vapors and gaseous elements [52,53,54,55], mitigating alternative substantial limitations. Prior to this study [56], a straightforward system verifying the sensitivity of the proposed device to smog, which impairs many material properties, was proposed. This section intends to evaluate the performance of the proposed sensor when subjected independently to temperature changes and CO_2_. Furthermore, a tangential approach regarding the multiple layers of the responsive substance has been adopted for the improvement of the sensing performance. Many specimens are tested to evaluate the reliability of reproduction. With regard to the selectiveness, the authors include the results of coating the first or topmost layer with a polymer-based ink for the sensitivity of the proposed sensor to CO_2_ and temperature. In the following subsection, we review the dimensions and design of a batteryless sensor device (see Figure 4), and explain its operating principles.

#### 2.3.1. Design and Principle

##### Radiation Mechanism of a Dual-Polarization Split-Ring Resonator (SRR)

**Figure 5:**
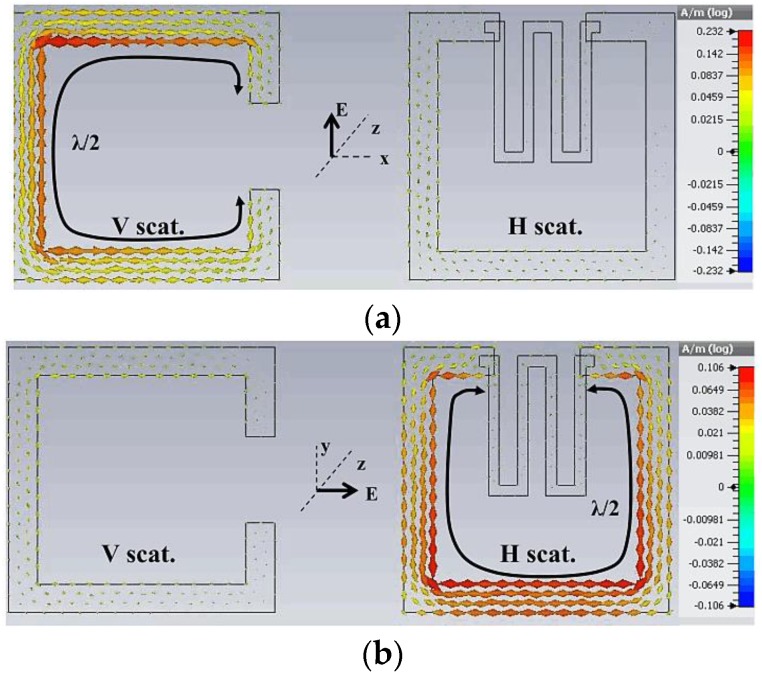
Electric-field distributions at 2.4 GHz for the dual-polarized SRR-based sensor: (**a**) for vertically polarized excitation; and (**b**) for horizontally polarized excitation [51].

The operational functionality of a batteryless RFID chip sensor device is comparable to the idea of a microchip-empowered RFID sensor without a unified analogue-to-digital converter (ADC). The observation of a dimensional criterion depends on the changes to the permittivity or conduction of a susceptible material. These variations result in the changes of the RCS of the RFID tag with respect to the frequency. Consequently, the magnitude shifts of some peaks and the resonant frequency can be sensed in the working range of the RFID tag. The electromagnetic device provides two different types of feedbacks on an equilateral basis. The electromagnetic (EM) results at one polarization are to be utilized for extracting the detected data, whereas the results at another polarization are to be utilized as a reference point for the identification of codes and calibration parameters. A similar idea was presented in [56] for smoke detection. Figure 5a,b shows currents in both scatterers when exposed to a vertically and horizontally polarized incident plane wave, respectively. From this illustration, it can be observed that only one scattering element is agitated at a particular polarization. Further, the EM response is isolated between each scattering element. Thus, variations in the degree of the detecting scatterer do not influence the degree of the corresponding referenced scatterer.

The physical sizes of the scattering elements have been adjusted to operate in the 2.4–2.5 GHz band. The SRRs are square-shaped with a lateral length of approximately 18 mm. The two arms of the SRR are 6 mm apart; the value of 6 mm is selected as a compromise considering the bandwidth of resonance, size, and the highest magnitude of the e-field current distribution. The gap of the SRR is maintained at 2 mm, allowing a low strip resistance, which is favorable for the magnitude of the RCS. Undeniably, a slimmer strip dimension might result in a degradation of the performance in terms of the conductivity of a strip lithographed using silver nanoparticle ink, comparatively smaller conductivity than the printed-circuit-board etching of copper. Figure 2a,b demonstrates a distance of 9 mm between the two scattering elements, allowing sufficient decoupling of the EM response (−15 dB isolation between polarizations). A minor estrangement space might have demanded higher bandwidth for the resonating dips in one and the other polarizations, also an abridged isolation of cross-polarization.

##### CNT Loaded Scattering Element for Sensing

A scattering element, which is denoted by “H” in Figure 4, will be used to detect information. An engraved patterning, which is composite SWCNT/poly(3,4-ethylenedioxythiophene) polystyrene sulfonate (PEDOT:PSS) ink-based [57,58], is interleaved between the space/gap of an SRR, to sensitize it to changes in various electrical parameters [59]. According to earlier characterizing processes, it is known that the conductivity and resolution of the deposit are the most sensitive parameters with regard to a temperature or gas variation. Consequently, a variable resistor can model the deposit. The impedance is at a maximum in the gap. Consequently, a strong deviation in the sensing response may be observed because in the case of CNT ink, the bridging resistance of the deposit has a high value. To increase the sensitivity of the sensing device, the size of the sensitive area has to be maximized. Conversely, to avoid canceling the dominant resonant frequency mode of the scattering element, the authors could not deposit a large resisting pattern in the SRR space. For such a structure, the resisting lining can be modeled as a resistor in parallel with a circuit of resonance. Thus, if the bridge resistance is minimized, the quality factor (QF) of the resonance dip is reduced. Consequently, a resonant peak cannot be detected at low bridging resistances. The sensitive stripline is inserted into the SRR gap, as illustrated in Figure 4, to prevent covering a majority of space. The aim was to determine the longest path covering the large area inside the space of an SRR. Further, the delicate stripline is in the shape of a meander line. The ratio of the length and width of the route is selected in such a way that minimal bridge resistance is accomplished with respect to the following research of sensitivity. Hence, a stripline width of 0.75 mm with a route length of 54 mm is employed. Furthermore, to ensure a healthy electrical connection between the SRR and sensitive stripline, the SWCNT/PEDOT:PSS-based stripline overlays with the silver stripline toward and adjacent to the gap-space, with a surface area of 4.5 × 2 mm^2^ (Figure 4).

In order to determine the minimum bridge resistance required to maximize the logarithmic and linear RCS changes, the authors performed a parametric simulation using CST Microwave Studio (CST MWS) by controlling the plate resistance of the susceptible stripline between 10 Ω/sq and 100,000 Ω/sq. They demonstrated a susceptible stripline area with zero thickness, which can be termed as an ohmic area in CST software.

**Figure 6:**
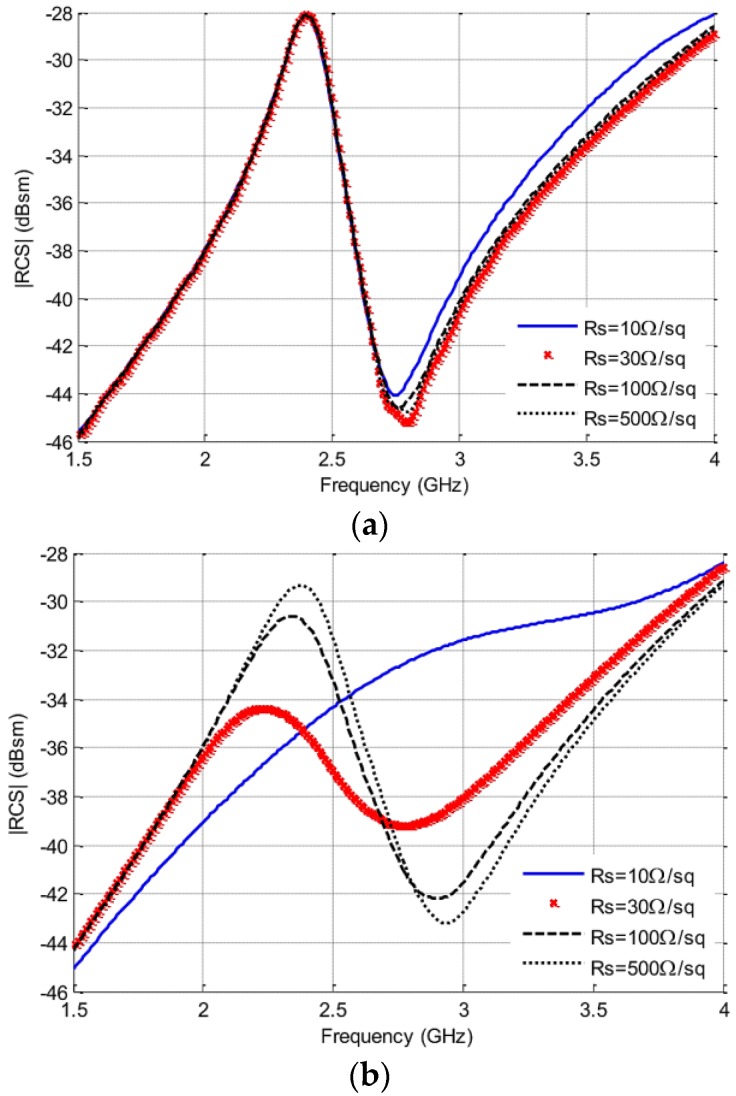
Simulated RCS responses as a function of the plate resistance of the SWCNT deposit for: (**a**) the vertical polarization; and (**b**) the horizontal polarization [51].

The simulation results for the RCS responses are illustrated in Figure 6a,b for several plate resistances, for both horizontal and vertical polarizations, respectively. Figure 5a shows the decoupling among both the polarizations, since by modifying the plate resistance of the susceptible deposit, no response was observed in the vertical polarization result. Instead, a noteworthy deviation was observed in the magnitude of the horizontal polarization result.

#### 2.3.2. Description of the Measurement Setup

**Figure 7:**
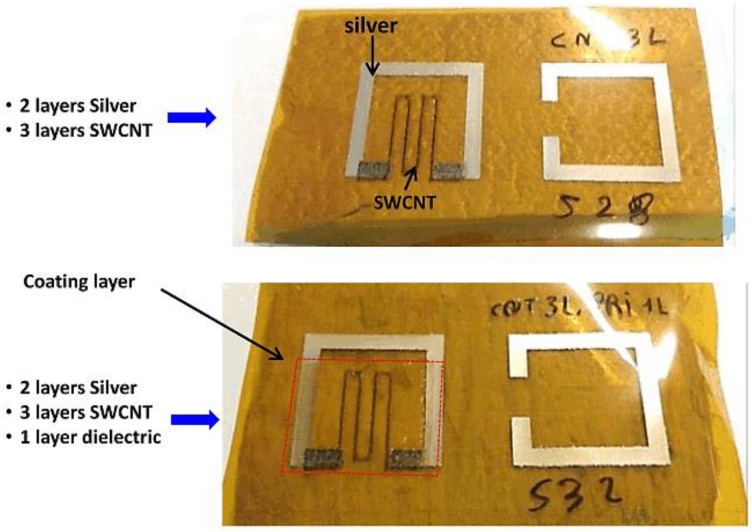
View of the fully inkjet-printed sensor on a polyimide material. The bottom figure contains an added see-through covering film on top of the susceptible deposit [51].

The sensors shown in Figure 7 are inkjet-printed over a stretchable 50-μm-thick polyimide coating used as a specimen. The permittivity of polyimide is 3.5 with a tangential loss value of 0.0027. The authors utilized the Dimatix DMP-2831 inkjet printer for the deposition of the material. They used a silver ink known as Harima Nanopaste for generating higher conductivity in the stripline. Two layers with a resolution of approximately 635 dpi (dots per inch) were printed initially, followed by a sintering process for 70 min at 130 °C to achieve the thickness of 2 μm. The plate resistance achieved was approximately 0.5 Ω/sq.

Further, the authors used composite SWCNT/PEDOT:PSS conducting ink for the susceptible conductive stripline [57,58]. The delicate material is inkjet-printed at a resolution of 1694 dpi after being cured at 30 °C for 30 min. The process of sintering is not compulsory, and the ink dries rapidly in the ambient atmosphere.

**Figure 8:**
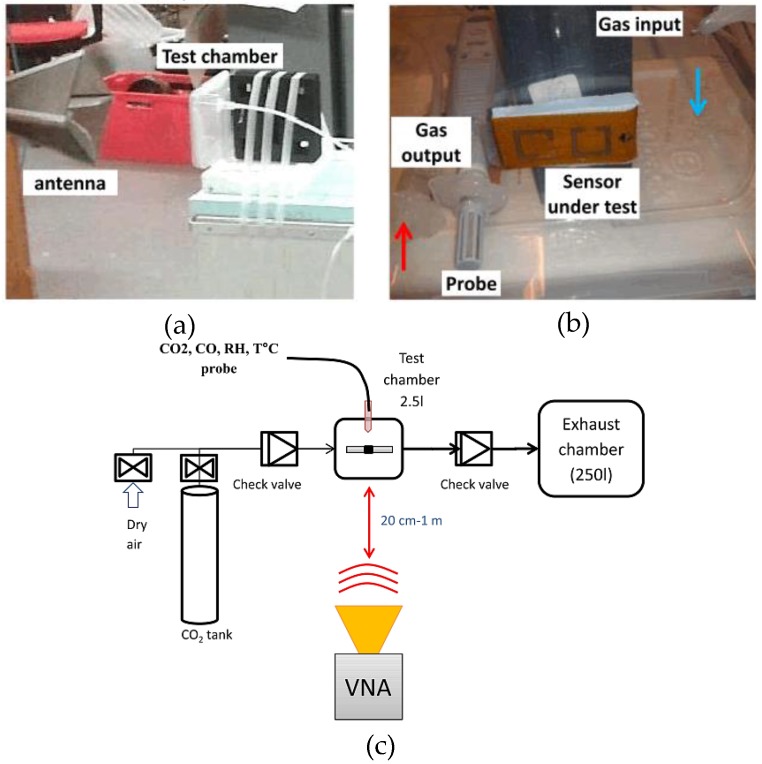
Experimental process: (**a**) view of the antenna in front of the test chamber; (**b**) view of the test chamber; and (**c**) description of the set-up for gas measurement [51].

The authors of [51] produced nine models of the prototype illustrated in Figure 4 and Figure 7. The dimensions of the susceptible strip-lines and the SRR were maintained exactly equal. However, the number of layers of the CNT-based stripline was varied between two and four. The measurement process in Figure 8 was utilized for carrying out the CO_2_ experiment. An airtight plastic rectangular box sufficiently large to enclose the sensing device, as shown in Figure 8b, was utilized as the device-under-test chamber. The testing box contained an inlet and an outlet with checked regulators to avoid any additional composite vapor travelling backwards. Dry air (10% relative humidity) or any specific gas were injected into the testing box. According to a sensitivity research of CNTs implemented in [52], numerous gases—e.g., NO_2_, NH_3_, and CO_2_—can be detected. In this subsection, we focus on the sensitivity of the SWCNT deposit for CO_2_ only. CO_2_ gas was injected using a quick hand-operated pump. Each injected amount soaks the purity of the gas inside the box at a degree of approximately 20,000 ppm. A HD37AB17D Delta Ohm probe recorded the purity of CO_2_, and simultaneously measured the humidity and temperature. An ETS-Lindgren 3164-04 wideband dual-polarized ridged horn antenna, with a gain between 9 dBi and 12 dBi and frequency in the range of 3 GHz to 6 GHz, was positioned 20 cm from the testing box. The terminals of this antenna were joined to an Agilent PNA E8358A vector network analyzer.

This subsection describes a stretchable inkjet-printed batteryless sensing device, and evaluates its sensitivity to CO_2_ gas and temperature [51]. An analysis to make this device reactive only for temperature variation is also presented. Wireless measurement of the device, imperiled to a CO_2_ purity of approximately 20,000 ppm, displayed changes of 0.23 dB and 0.51 dB with and without a dielectric covering, respectively. The authors also observed variations of the magnitude of approximately 2 dB in the changed prototypes over a temperature range of 35 °C to 65 °C. The top covering film in this research did not interfere with the temperature recordings of the sensing device.

## 3. 3D Printed Sensors

**Figure 9:**
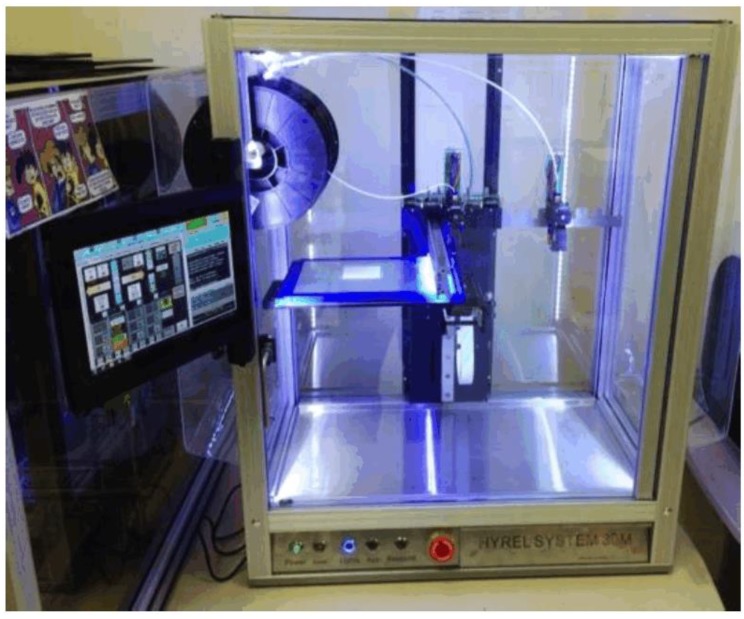
Hyrel System 30 3D printer [60].

3D printers have been in use for approximately 35 years. With 3D printing, objects are constructed brick-by-brick with comprehensive digital outlines [24]. Moreover, 3D printing has been utilized in fabrication. Free software available online and low-cost 3D printers and 3D printing supplies have resulted in the immense popularity of this trend. The benefits of 3D printing machinery include quick fabrication and the ability to construct difficult structures from more than one material, which is a limitation of the classic fabrication techniques. It has become evident that were will be a proliferation of 3D-printed applications in the near future. A Hyrel [60] System 30 3D printer is illustrated in Figure 9. The printer utilizes Repetrel, a revised variety of the Repetier controller software, which employed the mutual slicing computer-aided drawing software, known as Slic3r [61,62].

### 3.1. Novel Strain Sensor Based on 3D Printing Technology and 3D Antenna Design

The foremost 3D printed stretchable RF strain sensing device is discussed here [18]. The RF response of NinjaFlex, the famous 3D printer material, was characterized. A 3D antenna was analyzed and constructed using these materials and stretchable electrically conductive adhesives (ECAs). These materials hold immense promise for the upcoming 3D-printed RF solicitations, e.g., wearable RF components and flexible 3D sensing devices. The NinjaFlex filament was presented by Fenner Drives, Inc. in 2014 as one of the latest commercial 3D printing provisions [63]. NinjaFlex is a kind of thermoplastic elastomer (TPE) composed of thermoplastic and rubber [64]. The properties of TPEs hypothetically allow 3D printing to spread to various new domains, such as wearable antennas and RF electronics, owing to their elasticity and higher flexibility. Following its announcement, NinjaFlex was used in a variety of assignments [65,66]. This part of the review discusses a batteryless strain sensor based on 3D printing stretchable ECAs and NinjaFlex.

#### 3.1.1. 3D-Printed Strain Sensor Prototype

##### 3D Antenna Design

**Figure 10:**
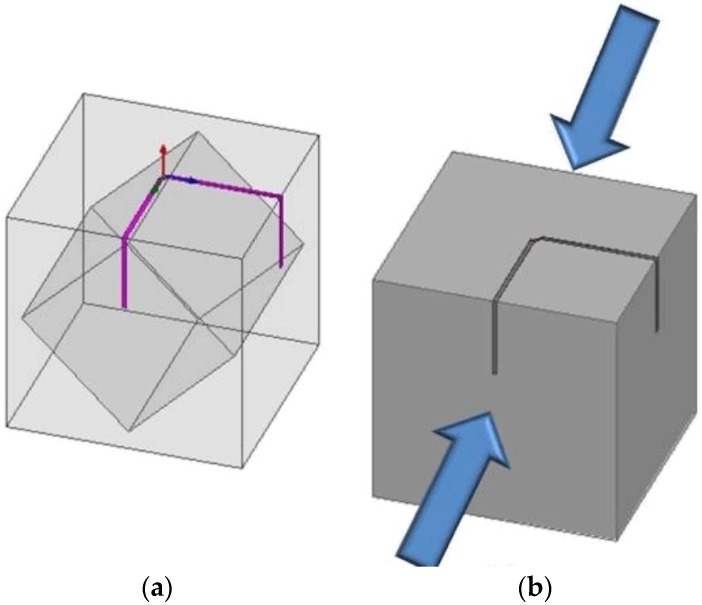
(**a**) 3D antenna on a hollow cube; and (**b**) directions in which strain is applied on the front and the back surfaces of the cube [18].

The dimensions of the dipole structure are shown in Figure 10. The dielectric material is a 30 mm × 30 mm × 30 mm hollow cube made of NinjaFlex (dielectric constant of 2.98, and tangential loss of 0.06 at 2.4 GHz). With two perpendicular arms, the dipole antenna is constructed using ECAs. We can observe in Figure 10a that the dipole is positioned on the top exterior of the hollow cube and is bent toward two additional planes on the sides. The feeding point of the dipole is at the midpoint of the top exterior. The two arms extend to the edge of the top surface and bend along the other two vertical surfaces. A similar dipole array geometry was also presented recently [67]. This 3D structure facilitates simple quantitatively analysis of the changes to antenna topology caused by strain. The NinjaFlex structure contains a hollow cube at its center. This structured design improves the quality of printed NinjaFlex, and enables easy stretching of the part of the dipole antenna on the front surface, as shown in Figure 10. The directions in which strain is applied can be observed in Figure 10b.

##### 3D-Printed Strain Sensor Prototype

**Figure 11:**
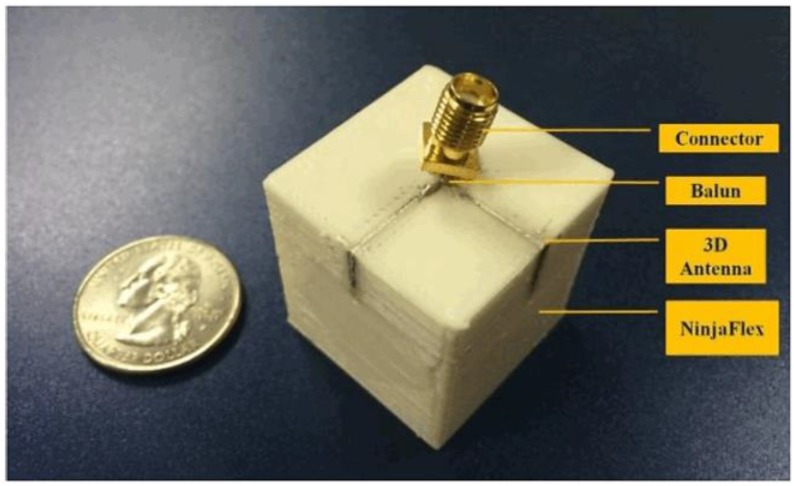
Ready-to-test 3D-printed strain sensor [18].

First, the cube and antenna traces were 3D-printed using a Hyrel System 30 3D-printer and NinjaFlex filament. Subsequently, the antenna traces were filled with the ECAs, based on a design in ANSYS High Frequency Structure Simulator (HFSS). An impedance-matched balun was added between the two antenna traces to connect to a sub-miniature version A (SMA) connector for insertion loss tests, as shown in Figure 11.

#### 3.1.2. Strain Experiment and Results

**Figure 12:**
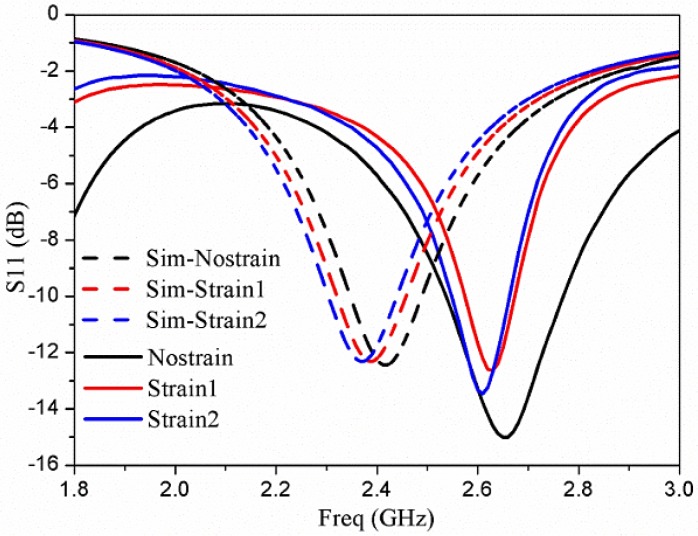
Results of measurements and simulations of the strain test of the 3D-printed strain sensor [18].

Strain was applied to the front and rear faces of the cube-shaped box (Figure 10b), and the change in the resonant frequency of an antenna was observed. Two dissimilar intensities of strain were applied to the box during experimentation. The measured results are illustrated by the solid lines in Figure 12. We observed that the center frequencies shifted by 30 MHz and 50 MHz, respectively, after applying strain-1 and strain-2. Owing to the 3D structure of the box, the most noticeable alteration in the length of the antenna is at the front. After applying a strain, this location of the structure stretches owing to the NinjaFlex. Consequently, the resonant frequency of the antenna is decreased as anticipated. In [18], a 3D antenna for use as a strain sensor was planned and constructed using stretchable ECAs and NinjaFlex. This technology offers enormous prospects for future 3D printing RF equipment, e.g., wearable RF devices and 3D stretchable sensor modules.

### 3.2. Microfluidic Sensor Constructed on a Flexible Material of Kapton for Measurement of Complex Permittivity of Different Liquid Materials

A sensitive and low-cost microfluidic sensor operating in the frequency range of 10–12 GHz was validated and proposed in a previous study [68]. This sensor contains various dabs coupled to a micro-strip (MS) line. The sensor is used for measuring and characterizing liquid chemicals, with applications in chemical laboratories and biological fields. The device is constructed on a stretching Kapton material by utilizing electronic printing technologies. With the help of mathematical calculations that describe the characteristics of resonance, the variance between the complex permittivity of a test and a reference sample predicts the complex permittivity of different concentrations of sodium chloride (NaCl) water solutions. The estimated numbers for the imaginary and real portions of the complex permittivity present a continuous deviation with the purity of the NaCl-water solution. Two of the linear zones correspond to the real part—one for minor concentration levels (<0.5 M), the other for larger concentration levels (>0.5 M). Only one linear region was achieved for the imaginary part for the several concentration levels examined. A close similarity is observed among the outcomes obtained from the Cole–Cole model and the experimental results recorded. The recorded experimental results determine the sensitivity and practicality of the addressed device for characterizing micro-quantity liquid chemical at microwave frequencies. Although the investigated frequencies were approximately 10 GHz, the same approach can be implemented for any frequency in the microwave range. Furthermore, the proposed method can be utilized to detect several new liquid chemicals that possess a complex permittivity at the same specifications of frequency using the same calibration. Although the proposed sensor is not common, it can be used favorably in a variety of practical experiments where the material under observation is a mixture or a liquid chemical.

#### 3.2.1. Design and Fabrication of the Liquid Chemical Sensing Device

##### Sensor Design

**Figure 13:**
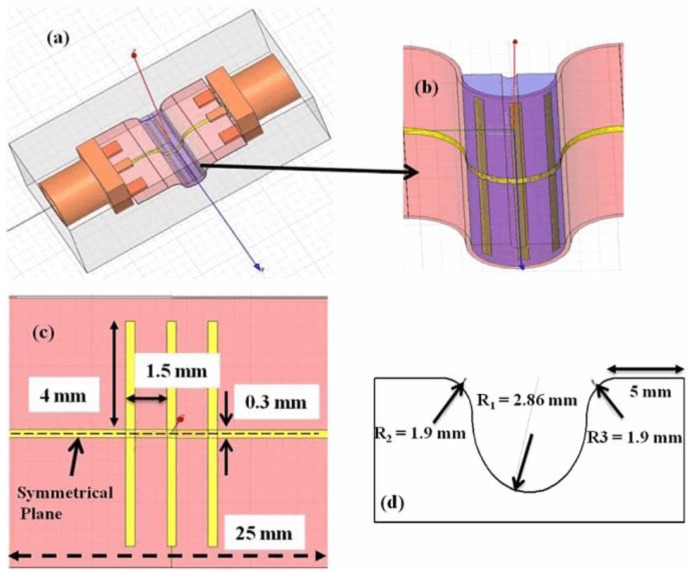
(**a**) Design and dimensions of the proposed sensor filled with aqueous chemicals; (**b**) zoomed structure of the bent portion highlighted in (**a**); (**c**) top view of the structure showing the dimensions; and (**d**) measurements of 3D-printed Acrylonitrile Butadiene Styrene (ABS) plastic module [68].

Figure 13 shows the design of the proposed RF chemical sensing device, constructed on 0.13-mm-broad Kapton material in HFSS. As evident from the illustration, this batteryless sensing device works on various dabs coupled to an MS line. The 25-mm-long MS has a width of 0.3 mm, as shown in Figure 13c, matching a characteristic impedance of 50 Ω. The size or length of every dab is 4 mm, improved for a 50-Ω impedance matching. Two of the dabs are located symmetrically opposite on each side of a central dab, which is situated at the center of the MS line. The space between the dabs is fixed at 1.5 mm. This sensing scenario is designed to have a larger detection space, where the E-field at resonance is also powerfully concentrated. A system is constructed for obtaining an accurate response in the X-band (8 to 12 GHz). The measurement parameters of the bent area are shown in Figure 13d. The sensor contains two orthodox segments (5 mm each) and three curves. With regard to the stab in the center, 9 mm of the substrate is bent at an angle of 180°, whereas the other two curves that match the two orthodox segments are curved from lengths of 3 mm at an angle of 90°.

##### Sensor Fabrication Process

**Figure 14:**
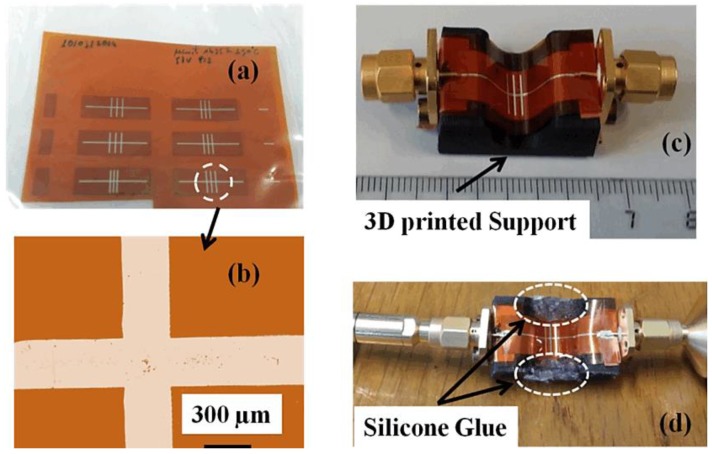
(**a**) Photograph of a set of resonators on Kapton substrate; (**b**) micrograph of a stub loaded along the MS line; (**c**) photograph of the sensor curved on a 3D-printed support; and (**d**) photograph of the sensor under testing [68].

The device is constructed on 130-μm-thick Kapton material using inkjet technology. Kapton was bought from Dupont Teijin films for this research. The reason for choosing Kapton material is its high thermal stability that enables sintering over high temperatures. Before printing, the Kapton sheet was washed in acetone chemical, cleaned by isopropyl alcohol, and completely dried using a flow of nitrogen. An easily accessible nanoparticle ink from market, acquired from Sun Chemical (Suntronic EMD5714) company, was used as a conducting material. The ink material contains silver nanoparticles distributed inside a blend of ethanol, glycerol, and ethanediol, at a 42% concentration by weight. Dimatix printhead (Spectra^®^ SE-128AA) was used for the deposition fixed in a Ceraprinter X-Series inkjet printer from Ceradrop society. The outlets were activated by a custom-made 57 V vibration at a jet frequency speed of 2 kHz. The space between the outlets and printing material was fixed at 800 μm. The drop spacing was fixed to 38 μm. Sintering was performed for 45 min at 200 °C for obtaining reliable silver tracker conductivity. The thickness of the final deposition material was as small as 1 μm for the metal parts (ground and conductive line). These were the constraints chosen to obtain acceptable realization with respect to conductivity (δ ≈ 5 × 10^6^ (S/m)) [69] while maintaining the physical and biochemical properties of the Kapton material unaffected. Figure 14a,b shows the pictures of the resonator prototype. Notably, the resolution of the conductive pattern formed by using inkjet-printing is acceptable and there were no wrinkles observed. The proposed microwave/microfluidic device is attached to SMA connectors with an overall 50Ω impedance matched at both the sides of the MS line as shown in Figure 14c. A 3D-printed mold was constructed using Acrylonitrile butadiene styrene (ABS) material and hardened at an oven temperature of 125 °C with measurements indistinguishable from those obtained in Figure 13. Silicone glue is attached to both sides of the bent device for avoiding seepage of the tested chemicals, as shown in Figure 14d. The Kapton material utilized here is easily curved on the 3D-printed mold and conforms to firm curves without the help of mechanical equipment.

#### 3.2.2. Simulation and Experiment Validity

**Figure 15:**
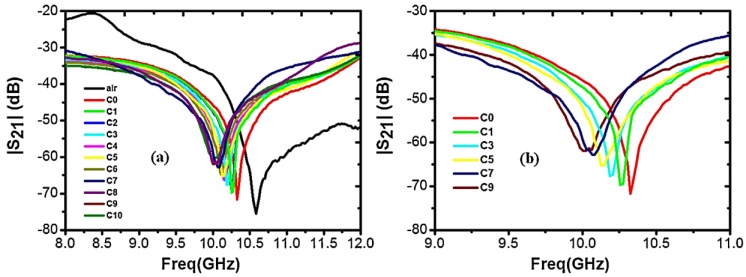
(**a**) Experimental *S*_21_ parameters of the sensor recorded in the X-band as a function of different concentrations of NaCl; and (**b**) zoom of (**a**), with selected concentrations to highlight the variation in the position of the resonant frequency and the magnitude of attenuation when the concentration is increased [68].

As previously mentioned, a mixture of deionized (DI) water with various purity mixtures of NaCl was examined to evaluate the sensitivity of the microwave/microfluidic device. The *S*_21_ spectrum illustrated in this paper was measured using a vector network analyzer (PNA-X N5242A (10 MHz–26.5 GHz)). Figure 15a presents the measured spectrum of the sensing device under experiment without and with the NaCl mixtures. In this study, the volume of the deposited solution was maintained constant (0.3 mL) for all concentrations. Notably, the recorded spectrum (*S*_21_) was completely repeatable with less than 0.29% change in the point of the resonant dip and approximately 1.9% variation in accordance with the magnitude of the attenuation dip. By considering the error generated by the depositor, the variations are also expected to escalate in a manner undefined yet. When there is nothing to examine (i.e., air is present on the sensor), the sensor has an insertion resonant dip of 76 dB at 10.61 GHz. When pure DI water (*C*_0_) is injected, it shifts the resonant dip to 10.32 GHz with an insertion loss of 70 dB. Notably, with the increase in the purity of NaCl in the mixture, the resonant dip shifts toward lower frequency values, and the bandwidth of the dips increases (Figure 15b). This outcome was expected owing to the fact that, when the purity of NaCl is increased, the real component of the complex permittivity is observed to decrease, resulting in a variation in the resonant dip. Furthermore, the decrease in the magnitude of the attenuation dip as the purity of NaCl in the mixture increases is clearly related to the increased magnitude of the imaginary component of the complex permittivity [70,71,72]. Simulations for many purity levels of NaCl were investigated manually using the HFSS computer tool for detecting the subtlest area of the device and for examining the magnitude of electric field distribution inside the bent region of the device during the experiment.

**Figure 16:**
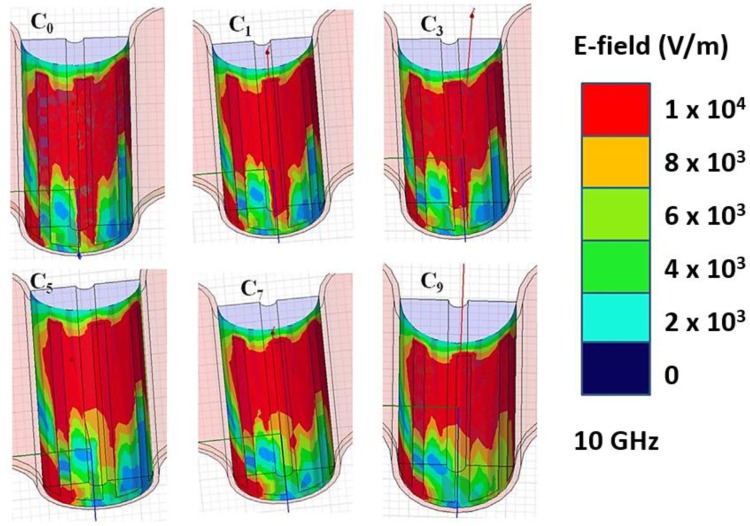
Electric field (E-field) distribution as a function of different NaCl-water concentrations calculated inside a symmetric half-structure of the sensor [68].

As shown in Figure 16, the half-structures of the sensor are simulated using symmetry (the symmetric plane is illustrated in Figure 13). The electric field distribution is evaluated at 10 GHz. In the case of C_0_, it is demonstrated that the electric field is intensely gathered at the center dab of the structure. Nevertheless, when the purity is increased, the electric field distribution is gathered toward the board of the dab, but not around the center dab, which is near the MS line, similar to *C*_0_. This result indicates that the center location close to the MS line is the most subtle with respect to the increment in the purity of the solution under test [68].

A cost-efficient microwave/microfluidic sensor that characterizes the complex permittivity of aqueous solutions in an efficient and exact manner is designed, fabricated, and validated in the X-Band. This sensor is realized using inkjet-technology and 3D printing on a Kapton substrate. Simulation and experimental investigations of the device are presented, and it is observed that the results obtained are consistent with the Cole–Cole model [68].

## 4. Screen Printing

Screen printing is a proven manufacturing technology that enables high-volume production at low cost. Thus, the main advantage of screen-printed RF sensors is the potential for low-cost and high-volume manufacturing. Practical implementation of screen printing technology for the fabrication of antennas began in the 1970s, when low-loss dielectrics arrived in the market. Screen printing technology offers the possibility for cost-optimized inline reel-to-reel manufacturing. Consequently, RF components can be thinner, lighter, more flexible, and cheaper than when fabricated using a conventional manufacturing process [73]. Screen printing is appropriate for fabricating electronics owing to the ability to produce patterned, thick layers from paste-like materials. This technique can produce conducting lines using inorganic materials (e.g., for circuit boards and antennas) and passive insulating layers, where the thickness of the layer is more important than the resolution. The characteristic throughput (50 m^2^/h) and resolution (100 μm) are similar to those observed with inkjet printing. This versatile and comparatively simple method is used primarily for the fabrication of conductive and dielectric layers [74,75]. However, organic semiconductors, e.g., organic photovoltaic cells [76] and complete organic field-effect transistors [77], can be printed.

### 4.1. Novel Strain Sensor Based on 3D Printing Technology and 3D Antenna Design

The design of a stretchable RF strain sensor fabricated using screen printing technology is suggested in [78]. The proposed sensing device is fabricated using a patch of half of the wavelength, which has a resonant frequency of 3.7 GHz. Its resonant frequency is determined by varying the size of the patch. Therefore, whenever the structure is stretched, it has a different resonant frequency. Polydimethylsiloxane (PDMS) was utilized as a substrate, since it is a stretchable and screen-printable surface. Dupont PE872 silver conductive ink (Dupont, NC, America) was utilized to produce a conducting structure with elasticity. The sensing operation is determined using full-wave computer simulations and experiments to be conducted on the fabricated prototype. After stretching, the resonant frequency of the device decreases to 3.43 GHz from 3.7 GHz, increasing the horizontal size by 7.8% and demonstrating a sensitivity of 3.43 × 10^7^ Hz/1%. When the device is stretched in the vertical direction, there is no change in the resonant frequency. 

#### 4.1.1. Design of a Strain Sensor

**Figure 17:**
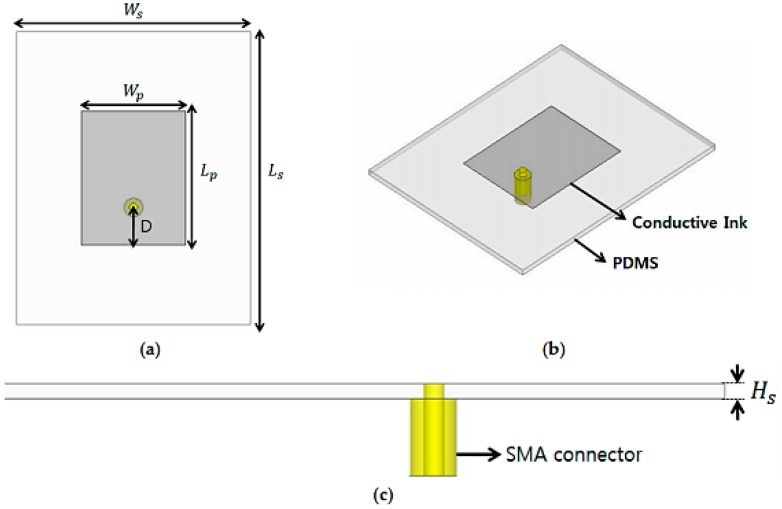
Dimensions of the addressed RF strain sensor: (**a**) top view; (**b**) perspective view; and (**c**) side view. *W_p_* = 17.05 mm, *L_p_* = 23.1 mm, *W_s_* = 40 mm, *L_s_* = 50.1 mm, *D* = 6.5 mm, and *H_s_* = 1.01 mm [78].

The construction of the sensing device is espoused from a rectangle resonator patch as shown in Figure 17. These four-sided patches are extensively utilized in RF resonators or structures containing resonator-based modules owing to their uncomplicated construction and ease of fabrication. In this particular research, the conductive patterning was generated using screen printing technology. The resistance of the surface of these conducting patterns was determined using the width-to-length ratio. The value of resistance for the silver conducting ink with stretching ability and the rectangle-shaped conductive patch are 0.64 Ω and 14.2 Ω, respectively. The diminished resistance on the surface of the rectangle-shaped patch is owing to the smaller width-to-length ratio. Figure 7 shows the dimensions of the strain sensor device. A coaxial transmission line feeding is utilized as an alternative to the classical MS line feeding. This modification is performed to ensure that the feed structure remains unchanged when the overall structure is stretched. Furthermore, PDMS is used here as a dielectric substantial or a substrate for the top conducting patch. The permittivity ($\varepsilon_{r}$) and tangential loss of PDMS were characterized using T-resonator process [79,80]. The resonant frequency ($f_{0}$) of the rectangle-shaped conducting patch can be computed as [81,82]:

(1) $$f_{0}=\;\frac{c}{2\sqrt{\varepsilon_{eff}}\left\{L_{p}+0.824H_{s}\left[\frac{\left(\varepsilon_{eff}+0.3\right)\left(\frac{W_{p}}{H_{s}}+0.264\right)}{\left(\varepsilon_{eff}-0.258\right)\left(\frac{W_{p}}{H_{s}}+0.8\right)}\right]\right\}}$$

(2) $$\varepsilon_{eff}=\;\frac{\varepsilon_{r}+1}{2}+\;\frac{\varepsilon_{r}-1}{2}\left[\frac{1}{\sqrt{1+12\left(\frac{h_{s}}{W_{p}}\right)}}\right],$$

where $\varepsilon_{eff}$ is the effective permittivity emanating from the fringing fields, c is the speed of light in vacuum, $W_{p}$ is the width of the rectangular patch, $L_{p}$ is the length of the rectangular patch, and $H_{s}$ is the thickness of the dielectric material (PDMS). The permittivity and tangential loss of the PDMS material were determined to be 3.01 and 0.025, respectively.

**Figure 18:**
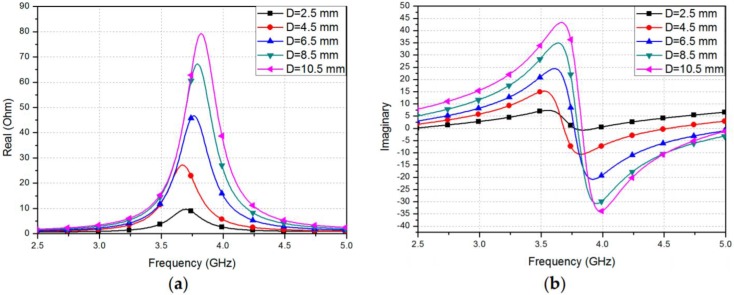
Simulated input impedance with *D* at different locations: (**a**) real part; and (**b**) imaginary part of impedance [78].

**Figure 19:**
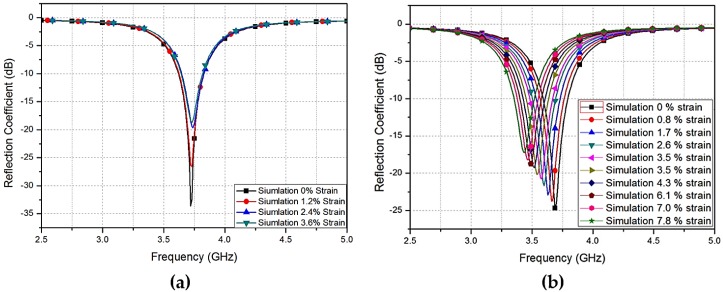
Simulated reflection coefficients with different patch: (**a**) widths; and (**b**) lengths [78].

The length and width of the rectangle-shaped conductive patch were selected to be 17.05 mm and 23.1 mm, respectively, to enable the sensor to have a resonant frequency of 3.7 GHz. Figure 18a,b displays the respective real and imaginary portions of the input impedance, with various values of *D*—the lengthwise distance of the coaxial feed to the edge of the rectangular patch. It can be observed in Figure 18 that the resonant frequency and impedance decrease with the increase in *D*. Thus, to realize matched impedance of 50 Ω, *D* is determined to be exactly 6.5 mm. ANSYS computer simulator (HFSS) is utilized for the full-wave simulation analysis. The SMA Version A connector was also incorporated in the simulation of the structure, as evident from Figure 17. If the resonant frequency highly depends on the varied lengths of the conducting patch, it is expected that the operating frequency will decrease when the device is to be stretched vertically. Figure 19a,b presents the simulation results of the reflection coefficient for different values of $L_{p}$ and $\;W_{p},$ respectively. Originally, the ideal sensor had a resonant frequency of 3.7 GHz with a reflection coefficient of −25 dB. The frequency of operation does not vary when $W_{p}$ is changed [83], as shown in Figure 19a. However, it is demonstrated in Figure 19b that the resonant frequency is reduced from 3.7 GHz to 3.43 GHz after stretching the device by 7.8% in the vertical direction. Consequently, the proposed device can be effectively utilized as a strain-sensing resonator by identifying variations in the resonant frequency. Therefore, to compute the elastic capability, the strain can be defined as:

(3) $$Strain=\;\frac{\Delta L}{L_{o}}\;\times 100\left(\%\right)$$

#### 4.1.2. Fabrication of the Strain Sensor

##### PDMS

**Figure 20:**
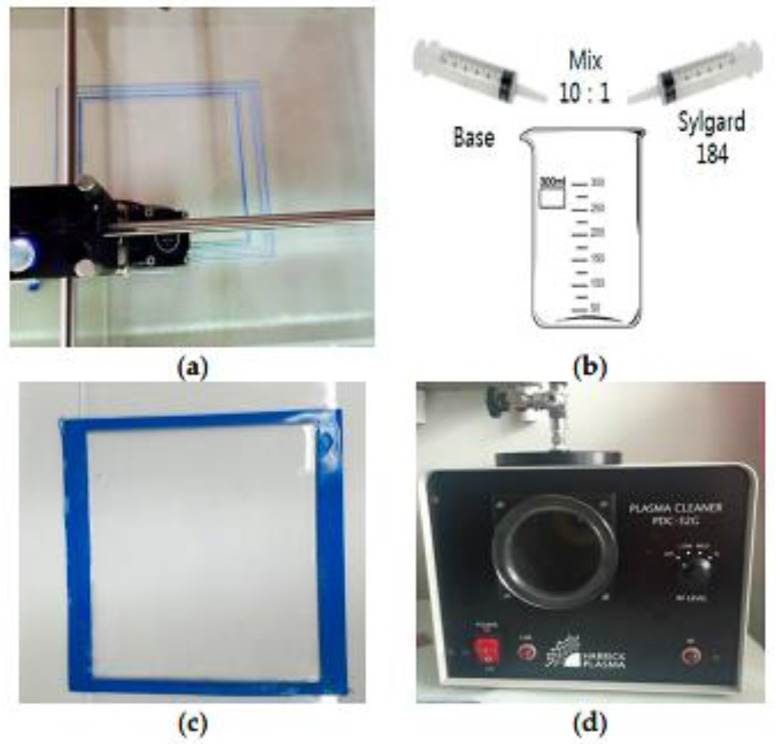
PDMS fabrication process: (**a**) 3D-printed mold for the PDMS substrate; (**b**) mixing the base and curing agent; (**c**) pouring liquid PDMS into the fabricated outline; and (**d**) plasma treatment processing [78].

Figure 20 presents the method to fabricate in-house PDMS material. In Figure 20a, the desired PDMS mold has been constructed on a plastic sheet using 3D printing (Ultimaker2 + 3D printer (Ultimaker B.V, Geldermalsen, The Netherlands)) procedure. Since 3D production is faster and easier than classic fabrication methods [84], it is extensively used. The thickness (*H_s_*), length (*L_s_*), and width (*W_s_*), of the flexible PDMS material were 1.01 mm, 50.1, and 40 mm, respectively. After constructing the mold, a liquid conformation of a fraternization, a curing agent with base was created with a ratio of 10:1. Subsequently, a void vacuum machine was utilized for the removal of air bubbles created during the fraternization process. A dense liquid conformation was cured at 30 °C for approximately 50 h, or at 110 °C for 40 min. The device also underwent heat curing process for 35 min at a temperature of 75 °C using a hot plate. Subsequently, a PDC-32G plasma cleaner (Harrick Plasma, NY, USA) was used to perform plasma treatment on the PDMS material. The plasma treatment was performed for approximately 20 s at 19 W.

##### Screen Printing

**Figure 21:**
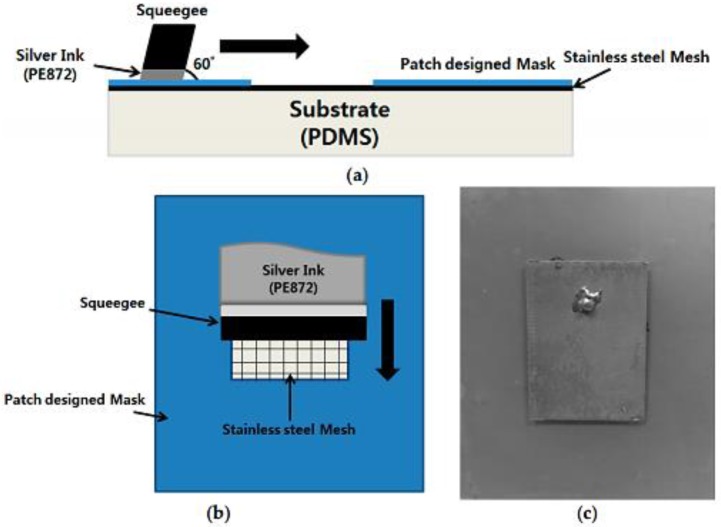
Silver screen printing process: (**a**) side view of screen printing process; (**b**) top view of screen printing process; and (**c**) picture of the fabricated prototype [78].

The silver screen printing method is shown in Figure 21. As shown in Figure 21a,b, the conducting pattern for the upper rectangle-shaped patch and the ground of the structure were screen-printed on the flexible PDMS material using the silver conductive ink (Dupont PE872, Bucheon, Korea), which exhibits elasticity. Daeyoung Technology Co. (Bucheon, Korea) produced the screen printer used in this project. The device has a printing speed in the range of 45–595 mm/s, and a squeegee angle between 60° and 90°. The 400 wire count mesh of stainless steel with a mesh tension of approximately 150 N was utilized. A mask of the pattern was created and placed on the PDMS material onto which the silver conducting nanoparticle ink was screen-printed using a squeegee. Figure 21c displays a fully fabricated and functional sensor. The rectangle-shaped conductive patch resides on the top, and the bottom is also screen-printed as the top patch. It is necessary to cure the prototype for improving the conduction on the screen-printed area. Therefore, heat sintering was performed in a vacuumed oven (ON-22GW) [85,86] for approximately 35 min at 90 °C. A hovel was subsequently pierced on the patch through to the bottom and an SMA connector pin was implanted using silver glue epoxy (the inner conductor pin of SMA attached to the top patch; the outer part of SMA connected to the ground in the same manner). 

#### 4.1.3. Experimental Results

**Figure 22:**
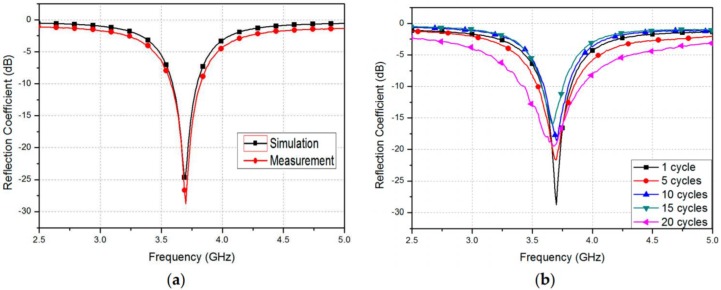
(**a**) Simulated and measured reflection coefficients of the proposed patch resonator before stretching; and (**b**) repeatability test of the fabricated strain sensor [78].

The specimen of the proposed RF sensor is shown in Figure 21c. Further, an Anritsu MS2038C vector network analyzer (Anritsu, Kanagawa, Japan) was used for the measurement of the reflection coefficients of the RF strain sensor. The measurement results of the reflection coefficients recorded for the strain sensor were compared with the corresponding simulation results in Figure 22a. Originally, the non-stretched device has a resonant frequency of 3.7 GHz with reflection coefficients of −27 dB. The recorded measurement results and the computer-simulated results are consistent with each other. The repeatability test is carried out for ensuring the reliability of results and is shown in Figure 22b. The graph shows the recorded reflection coefficient after every 1, 5, 10, 15, and 20 cycles. One cycle represents the relaxed state after stretching the strain sensor. It can be observed in Figure 22b that the resonant frequency does not change until 10 cycles of stretching and relaxation have been performed. The resonant frequency changes slightly to 3.67 and 3.68 GHz after 15 and 20 repetitions, respectively.

**Figure 23:**
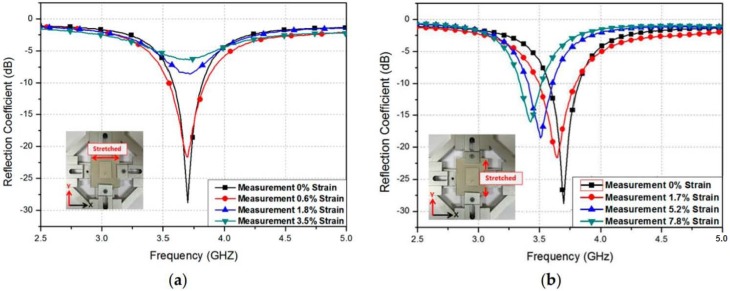
Measured reflection coefficients at different patch: (**a**) widths; and (**b**) lengths [78].

Furthermore, the reflection coefficients were recorded for different strain scenarios. The specimen was stretched along the vertical and horizontal axes; this can be observed in Figure 23. Figure 23a illustrates the recorded reflection coefficient results when the device is stretched along the vertical axis. It was already expected from the simulation results of reflection coefficients, as observed in Figure 19a, that the resonant frequency does not vary with the stretch being applied. Nevertheless, the impedance varies, since the coaxial feedhole becomes larger during the stretch. Moreover, Figure 23b demonstrates that the recorded reflection coefficient was changed as the stretch was applied in the horizontal direction. Compared to the reflection coefficient results of the simulation shown in Figure 19b, the resonant frequency varied from 3.7 GHz to 3.44 GHz, after a stretching of 7.82% was applied to the sensor. The strain of 7.82% corresponds to a 1.85 mm increase in length, and it was selected by maintaining the mechanical strength of the flexibility of PDMS material.

**Figure 24:**
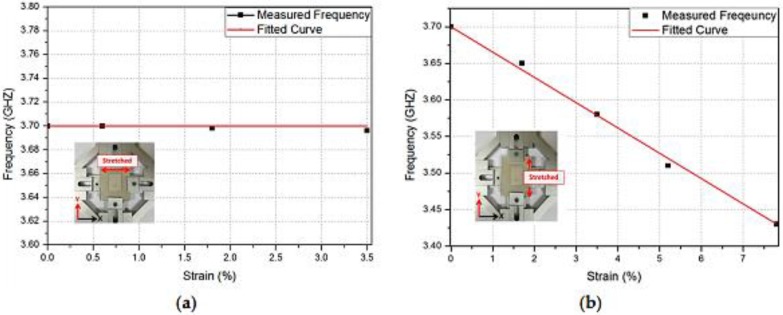
Relation between the resonant frequency and the strain along the: (**a**) *x*-direction (width); and (**b**) *y*-direction (length) [78].

The relationship between the resonant frequency and the strain alongside the vertical direction (width) and horizontal direction (length) is graphically shown in Figure 24a,b, respectively. As stated before, the frequency does not change when a strain is applied in the vertical direction; nonetheless, it is linearly proportional to the strain applied along the horizontal direction, as evident in Figure 24b. The sensor calibrate/fitting curve is defined as *y* = −0.0343*x* + 3.7. Consequently, the sensitivity of the proposed RF straining sensor is 3.443 × 10^7^ Hz/percentage. Table 1 confirms the significance of this work, and the proposed sensor is compared to the other strain sensors recently developed. The proposed RF strain sensor exhibits longer flexibility and strain scale owing to its stretch-proof conductive inks and flexibility.

In this subsection, an RF strain sensor using screen printing with stretch-proof silver conductive ink over a flexible PDMS material is presented. A rectangle-shaped top patch was considered and utilized for RF strain detection. The sensing operation considers the variation in the frequency of operation after a strain is applied along the vertical and horizontal dimensions of the sensing resonator. The practicality of the sensor was also verified by comparing both simulation and recorded results.

### 4.2. Flexible Screen Printed Biosensor with High-Q Microwave Resonator for Rapid and Sensitive Detection of Glucose

This section describes a sensitive and fast moderator-free glucose biosensor based on the phenomenon of an RF batteryless resonator realized using circular bent T-shaped identical impedance resonators printed using screen printing on a stretchy polyethylene material. Since the device has a high QF of 166, the proposed glucose biosensor has a sensitivity of 72 MHz/(mg∙mL^−1^) and an ultralow detection limit of 0.0167 μM at a central frequency of 11.8 GHz, within the linear detection range of 1–5 mg/mL. Moreover, the fair dependency of the loaded QF (*Q_L_*), propagation constant (*γ*), reflection coefficient (*S*_11_), and device impedance (*Z*) on the levels of glucose facilitates adequate multidimensional sensing by the glucose sensor.

#### 4.2.1. Designing and Construction of the Sensor

**Figure 25:**
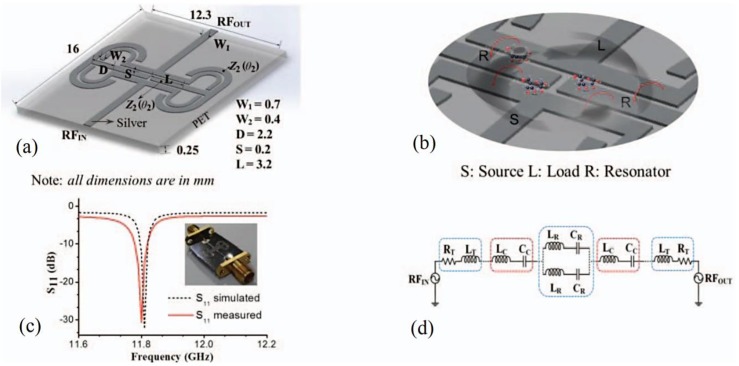
Proposed flexible glucose biosensor: (**a**) 3D layout of the resonator fabricated on a PET substrate; (**b**) schematic view of the sensing region of the resonator with a glucose–water solution; (**c**) simulated and measured *S*_11_, including a photograph of the fabricated resonator; and (**d**) the equivalent circuit of the proposed biosensing resonator [94].

Two circular-folded T-shaped uniform impedance resonators (TSUIRs) joined with parallel-coupled feeding lines for the construction of biosensor device over a PET complement are clearly illustrated in Figure 25a. The condition for the resonance of TSUIR is to be disclosed as an adjustment to the condition of resonance for the two sections of a stepped-impedance resonator, with *Z_in_* = 0 and *Z*_1_ = *Z*_2_ as below [91]:

(4) $$\tan\;\theta_{1}\tan\;\theta_{2}\;=\;\frac{Z_{2}}{Z_{1}}\;=\;1.$$

Figure 25a depicts two sections of the resonator with electrical lengths of $\theta_{1}$ and $\theta_{2}$. The measurements of the structure were optimized for resonance at the fundamental frequency of 11.8 GHz. This selection of frequency was relevant for glucose detection since the changes in the dielectric constant of this region are highly significant to the glucose–water solution with purity in contrast to the regions with lower frequency [92]. The geometry of the biosensing resonator compels the coupling gap (*S*) to affect the coupling coefficient significantly, which consequently affects the QF of the resonator [93]. Hence, the coupling gap was designated as the sensing region of the biosensor, as demonstrated in Figure 25b. The size of the coupling gap was optimized to 0.2 mm in order to realize a high QF of 166. Figure 25c shows a snapshot of the sensor prototype and compares the measurement and simulation results of *S*_11_ parameter of the resonator. The recorded fundamental frequency was reduced by 50 MHz. The quality factor was reduced, which was ascribed to a combination of bending loss, dielectric loss of the substrate, and physical dimension accuracy. Figure 25d demonstrates the corresponding design of the device loaded with a glucose section, expressed by *L_T_* and *R_T_*, which denote the source inductance and resistive loss of the load feed coupler, respectively. *L_R_* and *C_R_* represent the inductance and capacitance of the resonator, respectively.

*L_c_* and *C_c_* denote the inductance and capacitance, respectively, owing to the magnetic and electric coupling of the resonators with the load and the source, and are dependent on the glucose-level inside the testing sample. A Smart 3S screen printer was used to print the intended designs on a 0.245-mm-thick PET material using pg 007 silver ink (PURU, Seoul, Korea) diluted with ethylene glycol. The PET dielectric material has a permittivity of 3.1 and tangential loss of 0.0324. The deposited silver nanoparticle ink has a thickness of 1 μm. The printed patterns are placed in a heating chamber for 5 min at 150 °C to dry them and to increase the conductivity.

#### 4.2.2. Preparation of Testing Samples and Measurements

A standard solution of glucose consisting of a combination of DI water (Millipore TM) and D-glucose powder (SIGMA, life science, GC, St. Louis, MO, USA) was prepared at the concentrations of 1, 2, 3, 4, and 5 mg/mL. Subsequently, 2 μL of these liquid mixtures were positioned on the surface of the sensor using a micropipette. The reflection coefficients were recorded at frequencies ranging 1–15 GHz using an Agilent 8510C vector network analyzer. The testers were positioned over the detecting area of the biosensor after every 2 s.

#### 4.2.3. Detection using S-Parameters

**Figure 26:**
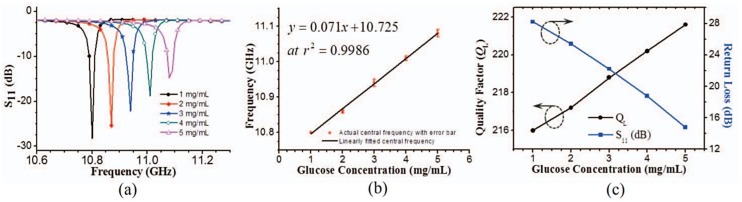
Detection of glucose level using measured S-parameters: (**a**) shift of central frequency; (**b**) linearly fitted central frequency and actual central frequency with error bar; and (**c**) variation of loaded quality factor (*Q_L_*) and return loss (*S*_11_) with variation in glucose concentration [94].

The shifts in the central frequency, indicated by the peak value of *S*_11_ of the five samples of glucose–DI water mixtures examined, are shown in Figure 26a. The fundamental frequencies of the sensor were 10.81 and 11.09 GHz for glucose samples with the maximum and minimum concentrations of 5 and 1 mg/mL, respectively. Therefore, the fundamental frequency of the sensor was observed to further reduce when the purity of the glucose liquid was reduced. However, for the rest of the glucose samples, the fundamental frequency increased from 10.81 GHz as the concentration of the glucose was increased. This behavior is caused by the interaction between aqueous glucose and the electromagnetic coupling among the resonators and feeding line. This interaction appears to be dependent on the increase in the permittivity of the glucose composition, as a result of the decrease in glucose concentration [95]. A regression analysis reveals a good linear correlation (*r^2^* = 0.9993) between the glucose concentration and shift in central frequency. The linear equation is expressed as follows:

(5) y=0.071x+10.725,

where *y* and *x* represent the central frequency and concentration of glucose, respectively. Therefore, the sensor exhibited a sensitivity of 71 MHz/(mg∙mL^−1^) for glucose–water solution. According to the optimization study and the associated calibration plot (see Figure 26b), the detection limit of the assay for a signal-to-noise ratio (S/N) of 3 was calculated as 0.0167 μmol of glucose in a 2 μL sample, as outlined in [96]. The S-parameters for each sample were measured four times. Although the points deviated from the central frequency, as shown by the error bars, there was no overlapping between each purity solution. Thus, the observation confirmed that the experiment is repeatable with the same phenomenon. Figure 26c shows the changes occurring in the loaded QF (*Q_L_*) and reflection coefficient (*S*_11_) of the sensor for glucose testing samples of different purity levels. *S*_11_ was maximized at −28.1 and −14.9 dB for glucose compositions of 1 and 5 mg/mL, respectively. There is a negative correlation between *Q_L_* and the concentration of glucose. This relationship is expected owing to the positive correlation between the loss factor and concentration of glucose.

#### 4.2.4. Detecting through Derived Parameters

**Figure 27:**
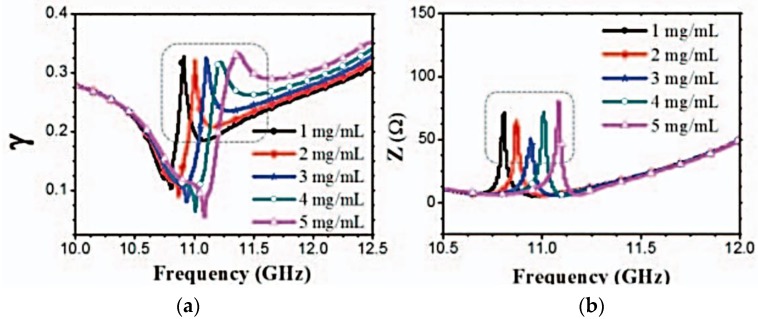
Detection of glucose level using derived parameters: (**a**) propagation constant (*γ*); and (**b**) impedance (*Z*) [94].

The propagation constant (*γ*) and impedance (*Z*) were obtained from the recorded reflection coefficients of the glucose testers, using the approach described in [97]. The propagation constant increased with changes in glucose purities, from approximately 11.51 GHz to 12.51 GHz, as shown in Figure 27a. Dips in resonance, which have a positive correlation between the frequency and glucose concentration, are observed. Dips in resonant impedance are also observed. These dips occur at dissimilar frequencies for varying glucose concentrations. The frequencies of these dips also have a positive correlation with glucose concentration, as shown in Figure 27b.

This subsection presents a stretchable biosensor using screen printing technology as a high QF RF batteryless resonator recognized for moderator-less sensing of glucose levels. Two circular-folded T-shaped uniform impedance resonators were joined with parallel-coupled feeding lines on PET sheets. Based on the central frequency shifts, the projected sensor device confirmed a highly sensitive and quick sensing mechanism of glucose with a significantly lower sensing limit.

## 5. Summary

Printed RF microelectronics is an evolving zone of investigation with larger commercial expectations owing to its capability to sidestep conventional inflexible and expensive silicon-based circuits to fabricate different types and shapes of components on bendable materials using high-quality printer methods. For the three additive techniques mentioned in this review, inkjet-printing is an alluring process for manufacturing electronic components owing to the negligible waste produced and its effectiveness at handling some expensive materials. Inkjet printing of the conducting forerunner materials, typically conductive nanoparticles or metallic–organic facilities, is employed as a comparatively faster method that can effectively handle roll-2-roll (R2R) manufacture. However, the sintering process in this fabrication, which is necessary to purify the patterns containing conductive inks, involves times longer than 20 min or higher temperatures (>200 °C). Specially, the higher sintering schedules are not scalable for R2R manufacturing. For instance, a web-speed of 1 m/s with a sintering time of 35 min corresponds to the production line required to be at least 1.9 km long. However, screen printing techniques are suitable for bulk production. Further, 3D printed structures, such as origami, are gaining interest owing to their ease of fabrication, which was previously an issue with the technique owing to the support structures required for fabrication. Some of the recent works involve combination of inkjet and screen printing in the development of batteryless sensors [98,99,100,101]. For this review, we have discussed and compared the recent advances in the three popular printing fabrication techniques with respect to their fabrication time, power consumption, and complexity. The focus was on the additive manufacturing of batteryless RF sensors and the advantages of these fabrication techniques for sensors perspective. 

## Figures and Tables

**Table 1:** Comparison of RF strain sensors [78].

	[78]	[87]	[88]	[89]	[90]
Substrate	PDMS	Kapton tape	Si	Duroid 5880	Kapton
Conductive Material	Au	Au	Au	Cu	Cu/Al
Maximum Strain (%)	7.8	N/A	N/A	0.2	1
Strain Gauge * (%)	7.3	5.69	0.21	0.14	2.35
Resonant Frequency (GHz)	3.7	12.3	0.4742	5	3.62

* Strain Gauge= Δff0 × 100(%)
